# Prolonged Effects of COVID-19-Related Behavioral Restrictions on Neonatal Bone Metabolism: A Retrospective Study

**DOI:** 10.7759/cureus.88618

**Published:** 2025-07-23

**Authors:** Tatenobu Goto, Ayako Uemura, Hiroto Maeda, Masashi Tanikawa, Mari Mizutani, Yuto Kondo, Megumi Suyama, Tomoki Goto, Kanemasa Maki, Katsumi Ushijima

**Affiliations:** 1 Division of Pediatrics, Yokkaichi Municipal Hospital, Yokkaichi, JPN

**Keywords:** covid-19 pandemic, maternal behavioral change, maternal-infant interaction, neonatal bone metabolism, newborn health, nutritional deficiency, pandemic impact, parathyroid hormone (pth), postnatal development, vitamin d deficiency

## Abstract

Objectives: The COVID-19 pandemic introduced significant lifestyle changes, such as reduced outdoor activities, social distancing, and other public health measures. These changes may have affected maternal health and neonatal biochemical parameters linked to bone metabolism. This retrospective study aimed to evaluate the impact of pandemic-related behavioral restrictions on neonatal biochemical markers, including calcium, phosphate, magnesium, intact parathyroid hormone (iPTH), and alkaline phosphatase (ALP), measured on the first day of life across three distinct time periods (pre-COVID-19, COVID-19 pandemic, and post-COVID-19).

Materials and methods: This retrospective cohort study included 964 neonates admitted to a Japanese neonatal intensive care unit between 2018 and 2024. Neonates were categorized into three groups: pre-COVID-19 (2018-2020), COVID-19 pandemic (2020-2022), and post-COVID-19 (2022-2024) periods. Serum biochemical parameters were measured on the first day of life and compared statistically among the groups.

Results: There were no significant differences in gestational age and birth weight among the three groups. Serum phosphate levels demonstrated a significant, stepwise increase across the periods (pre-COVID-19: 5.4 (4.8-5.9) mg/dL, COVID-19 pandemic: 5.6 (5.0-6.1) mg/dL, post-COVID-19: 5.7 (5.2-6.4) mg/dL; p<0.01). Magnesium levels significantly decreased in the post-COVID-19 period (pre-COVID-19: 2.05 (1.9-2.3) mg/dL, post-COVID-19: 1.90 (1.8-2.2) mg/dL; p<0.01). No significant changes were observed in calcium, iPTH, or ALP levels.

Conclusions: Pandemic-related lifestyle changes caused significant and persistent alterations in neonatal phosphate and magnesium metabolism, necessitating continuous monitoring and preventive strategies to address long-term health impacts.

## Introduction

Vitamin D deficiency (VDD) during pregnancy is a global health concern, affecting maternal and neonatal health worldwide. Despite the critical role of vitamin D in fetal skeletal development and maternal bone health, VDD remains prevalent due to various factors, such as inadequate sunlight exposure, dietary habits, and cultural practices [1].

Vitamin D plays a critical role not only in skeletal development but also in the maturation of the central nervous system. Its receptors are widely distributed in neurons and glial cells, and local synthesis within the brain suggests its importance for neurodevelopment [2]. Recent studies suggest that prolonged vitamin D supplementation during infancy may support motor and language development [3]. Conversely, maternal VDD during pregnancy has been linked to reduced head circumference and lower cognitive and language scores in offspring [4-6]. These findings highlight the influence of vitamin D status during pregnancy and early infancy on both bone health and brain development.

The prolonged global outbreak of COVID-19 profoundly impacted the lifestyles of pregnant women. Over an extended period, pregnant women were advised to adopt preventive measures, such as minimizing outdoor activities and practicing social distancing, to reduce the risk of infection. These behavioral changes, including reduced sunlight exposure due to lockdown restrictions, decreased physical activity, and alterations in dietary habits, may have significant implications for neonatal vitamin D status and bone metabolism during pregnancy. Calcium and vitamin D are critical for fetal skeletal development and bone health, and disruptions in their metabolism may adversely affect neonatal parathyroid function and bone metabolism at birth [7,8].

Several studies have highlighted the prevalence of VDD during pregnancy, particularly under pandemic-related restrictions, with reduced sunlight exposure playing a significant role in exacerbating the risk of vulnerable populations such as pregnant women [9]. Maternal VDD has been associated with altered neonatal calcium metabolism, impaired fetal bone mineralization, and changes in parathyroid hormone (PTH) levels [10]. However, the impact of prolonged maternal lifestyle changes during COVID-19 on neonatal bone metabolism and parathyroid function remains insufficiently explored.

This retrospective study aimed to evaluate the impact of COVID-19 pandemic-related behavioral restrictions on neonatal bone metabolism and parathyroid function. Specifically, we compared serum biochemical markers, including calcium, phosphate, magnesium, intact parathyroid hormone (iPTH), and alkaline phosphatase (ALP), measured on the first day of life among neonates born during three distinct periods: pre-COVID-19, COVID-19 pandemic, and post-COVID-19.

## Materials and methods

This retrospective study was conducted at the neonatal intensive care unit (NICU) of Yokkaichi Municipal Hospital, a comprehensive perinatal and maternal medical center located in Mie Prefecture, Japan. This study protocol was approved following the ethical standards approved by the Institutional Review Board of Yokkaichi Municipal Hospital (approval number: YMH-2024-9). All medical records used in this study were fully de-identified before analysis to ensure patient confidentiality. Neonates admitted to the NICU at our institution between April 1, 2018, and March 31, 2024, were included in this analysis. A total of 964 neonates met the inclusion criteria and were retrospectively reviewed using a consecutive sampling approach.

COVID-19 pandemic period and public health measures in Mie Prefecture

In response to the COVID-19 pandemic, the Japanese government implemented multiple public health measures, including a state of emergency and quasi-state of emergency (also known as “priority preventative measures”). The state of emergency involved nationwide behavioral restrictions, including requests to reduce outdoor activities, temporary closures of certain businesses, and limitations on public gatherings. The quasi-state of emergency was a more localized measure targeting specific regions, with actions such as reduced operating hours for businesses, restrictions on alcohol sales, and requests to avoid non-essential travel.

In Mie Prefecture, these measures were enforced during specific periods. The state of emergency was declared twice: first, from April 16, 2020, to May 14, 2020, and later, from August 27, 2021, to September 30, 2021. The quasi-state of emergency was implemented during three separate intervals: from May 9, 2021, to June 20, 2021; from August 20, 2021, to August 26, 2021; and from January 21, 2022, to March 6, 2022. Collectively, these measures spanned an approximately two-year period from April 16, 2020, to March 6, 2022. This period, referred to in this study as the COVID-19 pandemic period, was characterized by significant behavioral restrictions, which may have influenced maternal health behaviors and affected neonatal outcomes.

Study design and groups

The study spanned a total of six years, divided into three distinct two-year periods to evaluate the impact of the pandemic-related behavioral restrictions. The pre-COVID-19 period, defined as April 1, 2018, to April 15, 2020, represented the two years before the pandemic. The COVID-19 pandemic period, defined as April 16, 2020, to March 6, 2022, encompassed the two years during which public health measures were actively implemented. The post-COVID-19 period, defined as March 7, 2022, to March 31, 2024, represented the two years following the lifting of these restrictions. This three-period division allowed for comparisons to assess changes in neonatal parathyroid function during the pandemic and to evaluate any residual effects in the post-pandemic era.

Inclusion and exclusion criteria

Neonates admitted to the NICU during these periods were included in the study if they underwent serum iPTH testing on the first day of life. Cases were excluded if neonates were admitted after day 1 of life or had chromosomal abnormalities, multiple congenital anomalies, or severe neonatal asphyxia.

Data collection and statistical analysis

Serum levels of iPTH, calcium, phosphate, ALP, and magnesium were measured and compared among the three groups. Statistical analyses were performed using EZR software (Saitama Medical Center, Jichi Medical University, Saitama, Japan), which is a graphical interface for R (The R Foundation for Statistical Computing, Vienna, Austria) [11]. Normality of continuous variables was assessed using the Shapiro-Wilk test. Group comparisons were conducted using one-way analysis of variance (ANOVA) for normally distributed data or the Kruskal-Wallis test for non-normally distributed data. Bonferroni post hoc tests were used for pairwise comparisons where applicable. A p-value < 0.05 was considered significant. Results are presented as mean ± standard deviation (SD) or median (interquartile range (IQR)).

## Results

Study population

**Figure 1:**
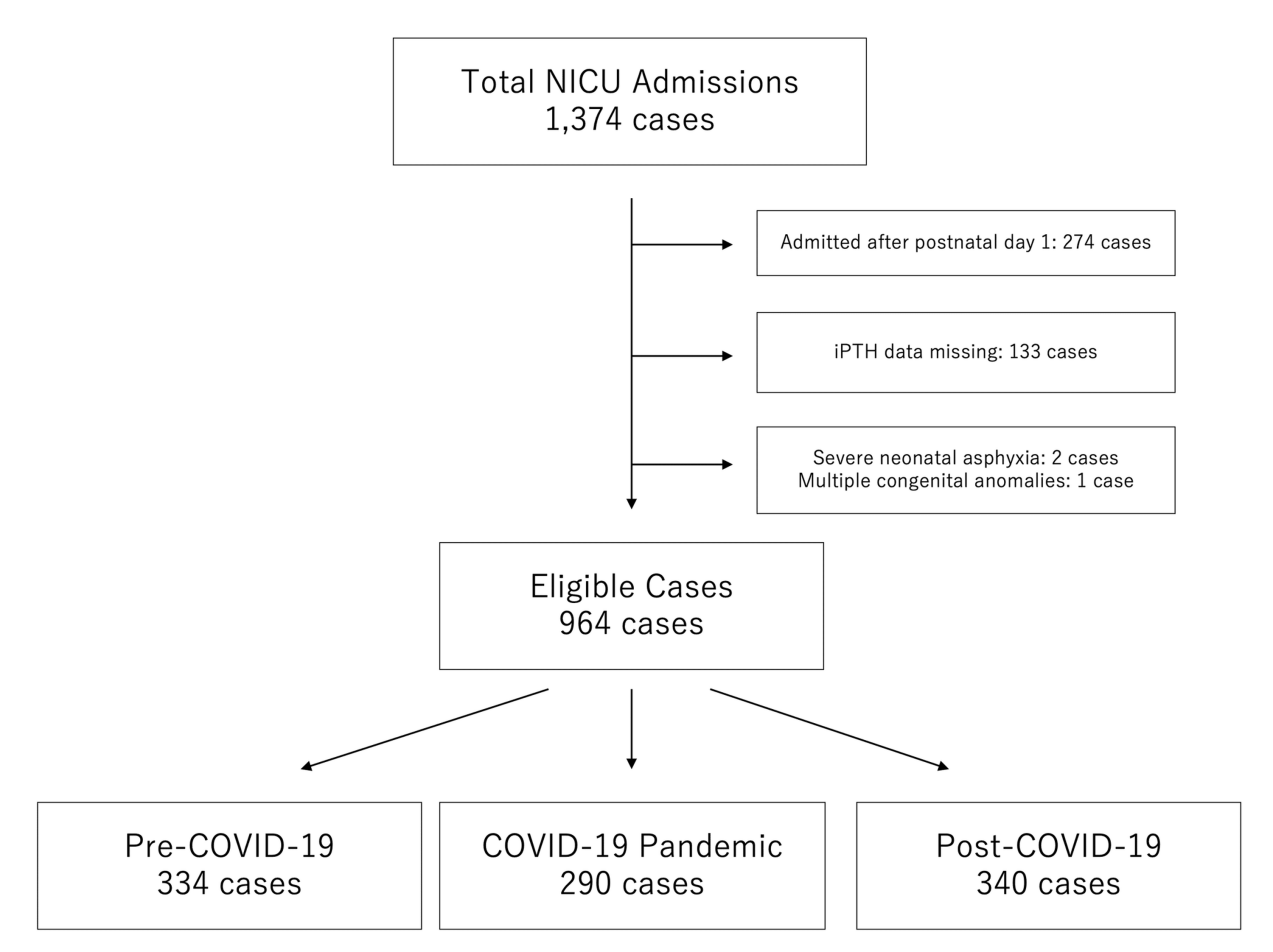
Study population and case selection criteria COVID-19: coronavirus disease 2019, NICU: neonatal intensive care unit

During the study period, a total of 1,374 neonates were admitted to the NICU. After applying the exclusion criteria, 964 cases were included in the final analysis. Excluded cases included 274 neonates admitted after day 1 of life, 133 neonates with missing data for iPTH, two neonates with severe neonatal asphyxia, and one neonate with multiple congenital anomalies. The included neonates were divided into three groups: 334 in the pre-COVID-19 period, 290 in the COVID-19 pandemic period, and 340 in the post-COVID-19 period (Figure 1).

Demographic characteristics

Analysis of demographic characteristics showed no significant differences in birth weight or gestational age among the three groups (Table 1). The mean birth weights were 2417 ± 770 g, 2413 ± 767 g, and 2490 ± 772 g for the pre-COVID-19, COVID-19 pandemic, and post-COVID-19 periods, respectively (p=0.11). Similarly, the mean gestational ages were 35.9 ± 3.7 weeks, 35.8 ± 3.5 weeks, and 36.0 ± 3.9 weeks, respectively (p=0.26).

**Table 1:** Demographic characteristics of neonates across the three study periods Gestational age (weeks) and birth weight (g) are presented as mean ± SD. Differences between groups were assessed using one-way ANOVA. ANOVA: analysis of variance, COVID-19: coronavirus disease 2019, N/A: not applicable (statistical comparisons were not performed), SD: standard deviation

Characteristic	Pre-COVID-19 period	COVID-19 pandemic period	Post-COVID-19 period	p-value
Number of cases (n)	334	290	340	N/A
Gestational age (weeks)	36.4 ± 3.6	36.3 ± 3.5	36.4 ± 3.9	0.33
Birth weight (g)	2415 ± 770	2413 ± 768	2490 ± 772	0.11

Biochemical parameters

All biochemical data and statistical analyses are summarized in Table 2.

**Table 2:** Biochemical parameters across the three study periods All data are presented as median (IQR). Differences between groups were evaluated using the Kruskal-Wallis test followed by Bonferroni-adjusted post hoc tests. Asterisks indicate statistical significance: *p<0.05; **p<0.01. Ca: calcium, P: phosphate, Mg: magnesium, iPTH: intact parathyroid hormone, ALP: alkaline phosphatase, IQR: interquartile range, ND: not determined in post hoc tests

Parameter	Pre-COVID-19 period (pre)	COVID-19 pandemic period (pandemic)	Post-COVID-19 period (post)	p-value	Post hoc test	
					(Bonferroni)	p-value
Ca (mg/dL)	9.6 (9.1-10.1)	9.6 (9.1-10.0)	9.5 (9.0-10.0)	0.204	ND	
P (mg/dL)	5.4 (4.8-5.9)	5.6 (5.0-6.1)	5.7 (5.2-6.4)	<0.01**	Pre vs. pandemic	0.034*
					Pandemic vs. post	0.043*
					Pre vs. post	<0.01**
iPTH (pg/mL)	16 (9-32.5)	12 (8-28.5)	15 (8-27.0)	0.071	ND	
ALP (U/L)	212.0 (181.0-260.0)	212.0 (176.5-257.8)	210.5 (174.0-261.0)	0.72	ND	
Mg (mg/dL)	2.05 (1.9-2.3)	2.00 (1.9-2.2)	1.90 (1.8-2.2)	<0.01**	Pre vs. pandemic	0.12
					Pandemic vs. post	0.048*
					Pre vs. post	<0.01**

Calcium Levels

Calcium levels remained stable throughout the three evaluated periods. The values were 9.6 (9.1-10.075) mg/dL in the pre-COVID-19 period, 9.6 (9.1-10.000) mg/dL during the COVID-19 pandemic period, and 9.5 (9.0-10.000) mg/dL in the post-COVID-19 period, with no significant differences (p=0.204).

Phosphate Levels

Phosphate levels exhibited a stepwise and significant increase across the three periods. The mean serum phosphate levels were 5.4 (4.8-5.9) mg/dL in the pre-COVID-19 period, 5.6 (5.0-6.1) mg/dL in the COVID-19 pandemic period, and 5.7 (5.2-6.4) mg/dL in the post-COVID-19 period (p<0.01). Pairwise comparisons confirmed that serum phosphate levels increased significantly between each pair of periods: pre-COVID-19 vs. COVID-19 pandemic (p=0.034), COVID-19 pandemic vs. post-COVID-19 (p=0.043), and pre-COVID-19 vs. post-COVID-19 (p<0.01).

iPTH Levels

The iPTH levels showed a downward trend across the three periods, with median values of 16 (9-32.5) pg/mL in the pre-COVID-19 period, 12 (8-28.5) pg/mL during the COVID-19 pandemic period, and 15 (8-27.0) pg/mL in the post-COVID-19 period. However, this decrease was not statistically significant (p=0.071).

ALP Levels

No significant differences in ALP levels were observed among the three groups. The median values were 212 (181.0-260.0) U/L in the pre-COVID-19 period, 212 (176.5-257.75) U/L during the COVID-19 pandemic period, and 210.5 (174.0-261.0) U/L in the post-COVID-19 period (p=0.72).

Magnesium Levels

Magnesium levels showed significant differences across the three periods (p<0.01). The median values were 2.05 (1.9-2.3) mg/dL in the pre-COVID-19 period, 2.00 (1.9-2.2) mg/dL during the COVID-19 pandemic period, and 1.90 (1.8-2.2) mg/dL in the post-COVID-19 period. Pairwise comparisons revealed significant differences between the pre-COVID-19 and post-COVID-19 periods (p<0.01; Table 2) and between the COVID-19 pandemic and post-COVID-19 periods (p=0.048).

## Discussion

This study highlights the long-term effects of COVID-19 pandemic-related behavioral restrictions on neonatal biochemical parameters, with notable alterations persisting into the post-pandemic period. Of them, the stepwise and significant increase in serum phosphate levels across the pre-COVID-19, COVID-19 pandemic, and post-COVID-19 periods is a novel finding. This observation suggests that maternal and neonatal metabolic disruptions, which likely began during the pandemic, have long-lasting consequences that extend beyond the immediate crisis. These findings emphasize the importance of addressing not only the acute impacts but also the prolonged effects of public health crises on maternal and neonatal health.

The COVID-19 pandemic has been associated with significant disruptions in maternal and neonatal health. Previous studies have reported that pandemic-related lockdowns significantly increased maternal and neonatal VDD, primarily due to reduced sunlight exposure and limited outdoor activities [9]. Moreover, disruptions in healthcare access and changes in dietary habits during the pandemic have been shown to contribute to maternal micronutrient deficiencies, with downstream effects on neonatal skeletal health and parathyroid function. Maternal VDD can result from lifestyle factors such as limited sun exposure due to clothing habits, insufficient dietary intake, and reduced outdoor activity, all of which can negatively affect maternal and neonatal calcium-phosphate metabolism [12].

Other studies have similarly documented the acute effects of behavioral changes, such as altered dietary habits and reduced physical activity, on maternal vitamin D status and its downstream impacts on neonatal calcium and phosphate homeostasis, as well as PTH [10]. In this context, the present finding of a significant stepwise increase in serum phosphate levels across the pre-COVID-19, COVID-19 pandemic, and post-COVID-19 periods aligns with prior reports indicating prolonged metabolic disturbances initiated during the pandemic.

The effects of maternal VDD on fetal serum phosphate levels remain underexplored, and no definitive conclusions have been established. However, several studies have reported elevated phosphate levels in neonates born to mothers with VDD, which is consistent with the findings of the present study [7,8]. In the context of maternal VDD, neonatal serum phosphate levels may increase due to impaired regulation by fibroblast growth factor 23 (FGF23).

Under physiological conditions, FGF23 suppresses phosphate reabsorption in the kidney and inhibits 1α-hydroxylase, thereby reducing 1,25(OH)₂D synthesis [13]. However, in cases of maternal VDD, decreased 1,25(OH)₂D may lead to lower FGF23 expression, as 1,25(OH)₂D is known to stimulate FGF23 production [14]. As a result, reduced FGF23 levels may lead to increased phosphate reabsorption in the neonatal kidney, resulting in elevated serum phosphate levels after birth. This mechanism is supported by previous findings showing that FGF23 suppresses phosphate reabsorption in the renal proximal tubules [15]. These results are in agreement with earlier studies that highlighted compensatory adaptations in neonatal calcium-phosphate metabolism in response to maternal VDD.

In addition, the transient decrease in magnesium levels observed during the pandemic may reflect both maternal dietary changes and physiological stress induced by pandemic-related restrictions, as suggested by earlier studies examining maternal nutrient profiles during public health crises [16]. This highlights the intricate relationship between maternal health behaviors and neonatal biochemical outcomes during unprecedented disruptions, such as the COVID-19 pandemic.

By integrating these findings with the broader literature, the present study underscores the need for targeted interventions to mitigate the long-term impacts of public health crises on maternal and neonatal health. Future research may investigate the mechanisms underlying these metabolic disruptions and evaluate preventive strategies, such as vitamin D supplementation and enhanced maternal nutritional support, during pandemics and similar crises. The observed stability in serum calcium levels across all three periods aligns with earlier research, suggesting that clinical interventions effectively maintained calcium homeostasis during the pandemic [8].

Even in cases of maternal VDD, fetal calcium levels may be maintained through efficient placental calcium transfer mechanisms. The placenta may actively transport calcium from the maternal circulation to the fetus, supporting fetal skeletal development. This process is thought to be primarily supported by maternal bone demineralization rather than maternal vitamin D status, suggesting that the fetus can receive sufficient calcium even if maternal stores are depleted [17].

However, the transient decrease in magnesium levels during the COVID-19 pandemic period, followed by recovery in the post-COVID-19 period, adds a new dimension to our understanding of neonatal metabolic adaptations during public health crises. The downward trend in iPTH levels, although not significant, is consistent with previous findings that maternal VDD can affect neonatal parathyroid function [10]. However, the lack of significant differences in iPTH levels across periods suggests that compensatory mechanisms have mitigated potential disruptions.

Vitamin D promotes calcium absorption in the intestines. When vitamin D is deficient, intestinal calcium absorption decreases, and PTH is secreted to compensate. Even if the mother tends to VDD, the fetus may receive enough calcium from the mother through the placenta as long as maternal calcium levels are sufficient. In such cases, additional PTH secretion in the fetus may be suppressed, leading to lower PTH levels.

A key methodological strength of this study is its ability to minimize the confounding effects of seasonal variation in iPTH levels, a phenomenon well-documented in prior research [18]. By dividing the study into approximately two-year intervals, this design ensures robust comparisons across periods while reducing the influence of seasonal fluctuations.

Furthermore, the stability of ALP levels throughout the study period may be attributed to stable placental calcium transfer, fetal compensatory mechanisms, and the limited influence of maternal nutrient deficiencies on ALP expression. Further, routine maternal supplementation and clinical care during pregnancy may have contributed to maintaining consistent neonatal bone metabolism.

Limitations

This study has several limitations that warrant consideration. First, its retrospective design introduces inherent biases, such as dependence on existing medical records, which may compromise the completeness and accuracy of the data. Second, the study was conducted at a single center within a specific geographical and cultural context, which may limit the generalizability of the findings to other populations or regions with differing healthcare practices or pandemic responses. Third, maternal data, including vitamin D levels, dietary intake, sunlight exposure, and socioeconomic status, were not collected. These factors are essential for understanding the mechanisms underlying the observed neonatal biochemical changes. Due to the absence of these data, no adjustments or stratified analyses could be performed, and the influence of these factors cannot be entirely excluded, which limits the ability to interpret the findings comprehensively. Fourth, although this study focused on neonatal biochemical parameters on the first day of life, it did not evaluate longer-term outcomes, such as bone development or metabolic health in later infancy or childhood.

Finally, though the study design effectively mitigated the effects of seasonal variation in iPTH levels, other potential confounding factors, such as disparities in maternal healthcare access or changes in clinical practices during the pandemic, could not be fully controlled. Future studies are encouraged to collect maternal vitamin D levels and dietary data to elucidate the mechanisms underlying neonatal biochemical changes better. Addressing these limitations in future research will be essential to validate the findings and further elucidate the long-term effects of pandemic-related behavioral changes on maternal and neonatal health.

## Conclusions

This retrospective study demonstrated that biochemical alterations in neonatal serum phosphate and magnesium levels associated with COVID-19 pandemic-related behavioral restrictions on neonates extend beyond the immediate pandemic period, with significant alterations in serum phosphate levels persisting into the post-pandemic era. These findings underscore the need for sustained attention to maternal and neonatal health during and after public health crises. Future research is warranted to elucidate the mechanisms underlying these prolonged effects and explore their implications for long-term neonatal health outcomes.
